# Mammographic radiomics and breast density for predicting PD-L1 expression in breast cancer

**DOI:** 10.1186/s40644-026-01001-3

**Published:** 2026-02-07

**Authors:** Yi-shan Zhao, Hao Li, Can-can Zhao, Yu-heng Wang, Ping Wang, Zong-yu Xie, Yu Ji, Hong Lu

**Affiliations:** 1 Department of Breast Imaging, Tianjin Medical University Cancer Institute and Hospital, National Clinical Research Center for Cancer, Key Laboratory of Cancer Prevention and Therapy, Tianjin, Tianjin’s Clinical Research Center for Cancer, Key Laboratory of Breast Cancer Prevention and Therapy, Tianjin Medical University, Ministry of Education, Tianjin, 300060 China; 2 Hangzhou Smart Intelligent Co. Ltd., Hangzhou, 311121 China; 3 Department of Radiology, The First Affiliated Hospital of Bengbu Medical University, Bengbu, Anhui 233004 China

## Abstract

**Background:**

Programmed death-ligand 1 (PD-L1) expression is a critical biomarker for guiding immunotherapy in breast cancer, particularly in triple-negative subtypes. However, conventional assessments rely on invasive biopsies and are limited by tumor heterogeneity. This study aims to develop a non-invasive approach for predicting PD-L1 expression using mammography-based radiomics features integrated with clinicopathological variables and breast density, and to evaluate its performance in both an internal development cohort and an independent external validation cohort.

**Methods:**

A total of 121 patients with breast cancer who underwent PD-L1 testing were retrospectively included, comprising 81 patients from Tianjin Medical University Cancer Institute & Hospital (development cohort, April 2023–September 2024) and 40 patients from the First Affiliated Hospital of Bengbu Medical University (external test cohort, January 2019–March 2025). Lesion regions of interest (ROIs) were manually annotated on both mediolateral oblique (MLO) and craniocaudal (CC) views for radiomic feature extraction using the SIMPACS Research platform. Additionally, standardized 1.5 cm × 1.5 cm ROIs were placed in the retroareolar parenchyma of both ipsilateral and contralateral breasts to evaluate background breast density. A multilayer perceptron (MLP) classifier was trained in the development cohort by combining lesion radiomic features, ipsilateral breast density radiomics, and clinicopathological variables, and then applied without recalibration to the external cohort. Performance was assessed using area under the receiver operating characteristic curve (AUC), accuracy, precision, recall, and F1 score.

**Results:**

In the development cohort, the radiomic model incorporating clinicopathological information achieved an AUC of 0.610. When ipsilateral breast density was added, the AUC improved to 0.731. In contrast, the models with contralateral and bilateral density achieved lower AUCs of 0.535 and 0.537, respectively. In the independent external cohort, the final ipsilateral radiomics–clinical MLP model achieved an AUC of 0.629.

**Conclusion:**

Mammography-based radiomics models may offer a non-invasive approach to predicting PD-L1 expression in breast cancer. The inclusion of ipsilateral breast density improves predictive performance and could support individualized immunotherapy decision-making. The observed performance in an external cohort provides preliminary evidence of cross-institutional generalizability, while highlighting the need for further optimization and validation in larger multicenter studies.

**Supplementary Information:**

The online version contains supplementary material available at 10.1186/s40644-026-01001-3.

## Introduction

Among women, breast cancer is the most commonly diagnosed cancer, and the leading cause of cancer deaths globally [1]. One key prognostic factor is its intrinsic molecular subtype [2].Triple-negative breast cancer (TNBC), defined by the lack of estrogen receptor (ER), progesterone receptor (PR), and human epidermal growth factor receptor 2 (HER2), accounts for 15%–20% of cases. TNBC is more aggressive, with higher rates of recurrence and poorer outcomes [3]. Its management is challenging due to the lack of targeted therapies, making chemotherapy the standard treatment [4].

Immune checkpoint inhibitors (ICIs) targeting the PD-1/PD-L1 pathway have demonstrated efficacy in TNBC, with PD-L1-positive patients often achieving better therapeutic outcomes [5, 6]. The Combined Positive Score (CPS), which accounts for PD-L1 expression in tumor and immune cells, is widely used to quantify PD-L1 status [7, 8]. Patients with higher CPS values (≥ 10) have shown improved responses to ICIs [9], underscoring its clinical utility in guiding treatment decisions.

Breast density, a well-established risk factor for breast cancer, has also been associated with tumor microenvironment characteristics and molecular markers [10, 11]. Evidence suggests that denser breast tissue is linked to elevated inflammatory markers, potentially supporting immune evasion mechanisms [12]. Currently, there is no conclusive evidence establishing a direct association between breast density and PD-L1 expression.

Conventional PD-L1 assessment via IHC-based CPS is invasive and limited by tumor heterogeneity and sampling bias. Radiomics offers a non-invasive alternative by extracting quantitative features from mammography images [13–16]. Incorporating breast density information may improve the prediction of PD-L1 expression and CPS by providing additional context about the tumor microenvironment. This study aims to develop a mammography-based radiomics model that incorporates breast density to predict PD-L1 expression.

## Materials and methods

### Study population

**Fig. 1:**
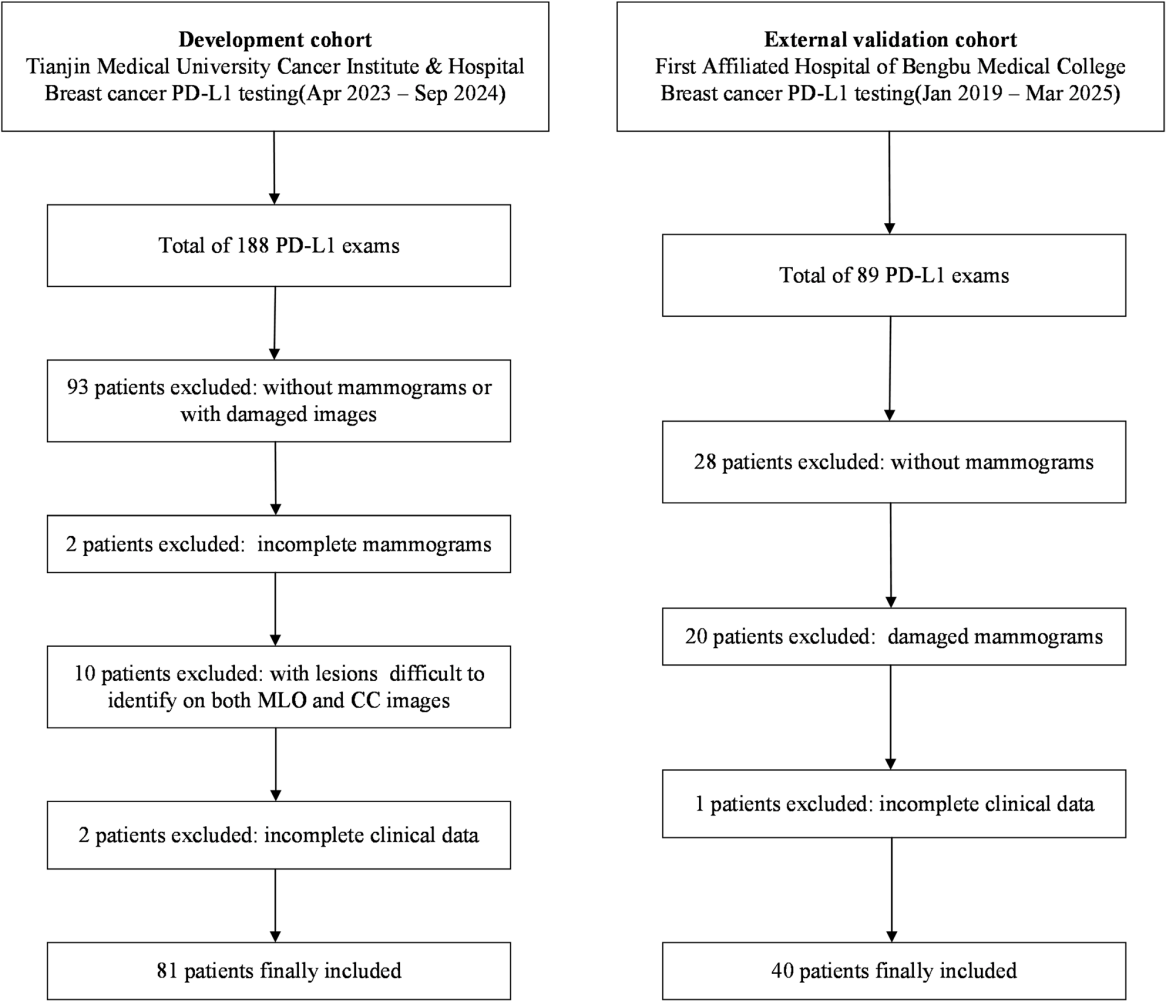
Flowchart of patient selection for the development and external validation cohorts

Due to the retrospective study design, the requirement for informed consent was waived. All consecutive breast cancer patients who underwent PD-L1 testing between April 2023 and September 2024 at Tianjin Medical University Cancer Institute & Hospital were retrospectively screened, and 188 patients were initially identified. Exclusion criteria were: (1) absence of pre-treatment mammography or mammograms that were damaged or non-diagnostic (*n* = 93); (2) incomplete mammographic views, such as missing either the MLO or CC projection (*n* = 2); (3) lesions insufficiently visible for reliable identification on both MLO and CC images (*n* = 10); and (4) incomplete clinical information (*n* = 2). After these exclusions, 81 patients were included in the development cohort and used to evaluate the relationship between mammographic radiomics features and PD-L1 expression (Fig. 1).

For external validation, an independent cohort was obtained from the First Affiliated Hospital of Bengbu Medical University. A total of 89 breast cancer patients who underwent PD-L1 testing between January 2019 and March 2025 were retrospectively screened. Patients were excluded if pre-treatment mammography was unavailable (*n* = 28), mammographic images were damaged or of insufficient quality for radiomics analysis (*n* = 20), or clinicopathologic data were incomplete (*n* = 1). Ultimately, 40 patients were included in the external validation cohort, which served as an independent test set to assess the generalizability and robustness of the final radiomics model.

### Combined Positive Score (CPS) testing

PD-L1 expression was evaluated using the 22C3 monoclonal antibody (Dako) on the DAKO Link 48 Autostainer platform, an FDA-approved companion diagnostic, which ensures standardized immunohistochemical staining and reproducible results [17]. The 22C3 assay’s reliability for CPS evaluation in metastatic triple-negative breast cancer (mTNBC) has been validated in studies like the KEYNOTE trials, where CPS ≥ 10 identified patients likely to benefit from pembrolizumab [18]. Furthermore, 22C3 has demonstrated robust interobserver reproducibility, reinforcing its clinical utility for guiding immune checkpoint inhibitor therapy [19].

### Pathological evaluation

We obtained histopathological information including tumor grade as well as ER, PR, HER2, and Ki-67 status through medical record systems. Threshold values were ≤ 1% for ER and PR levels and ≤ 14% for Ki-67 [20].

### Breast lesions and parenchymal patterns segmentation

**Fig. 2:**
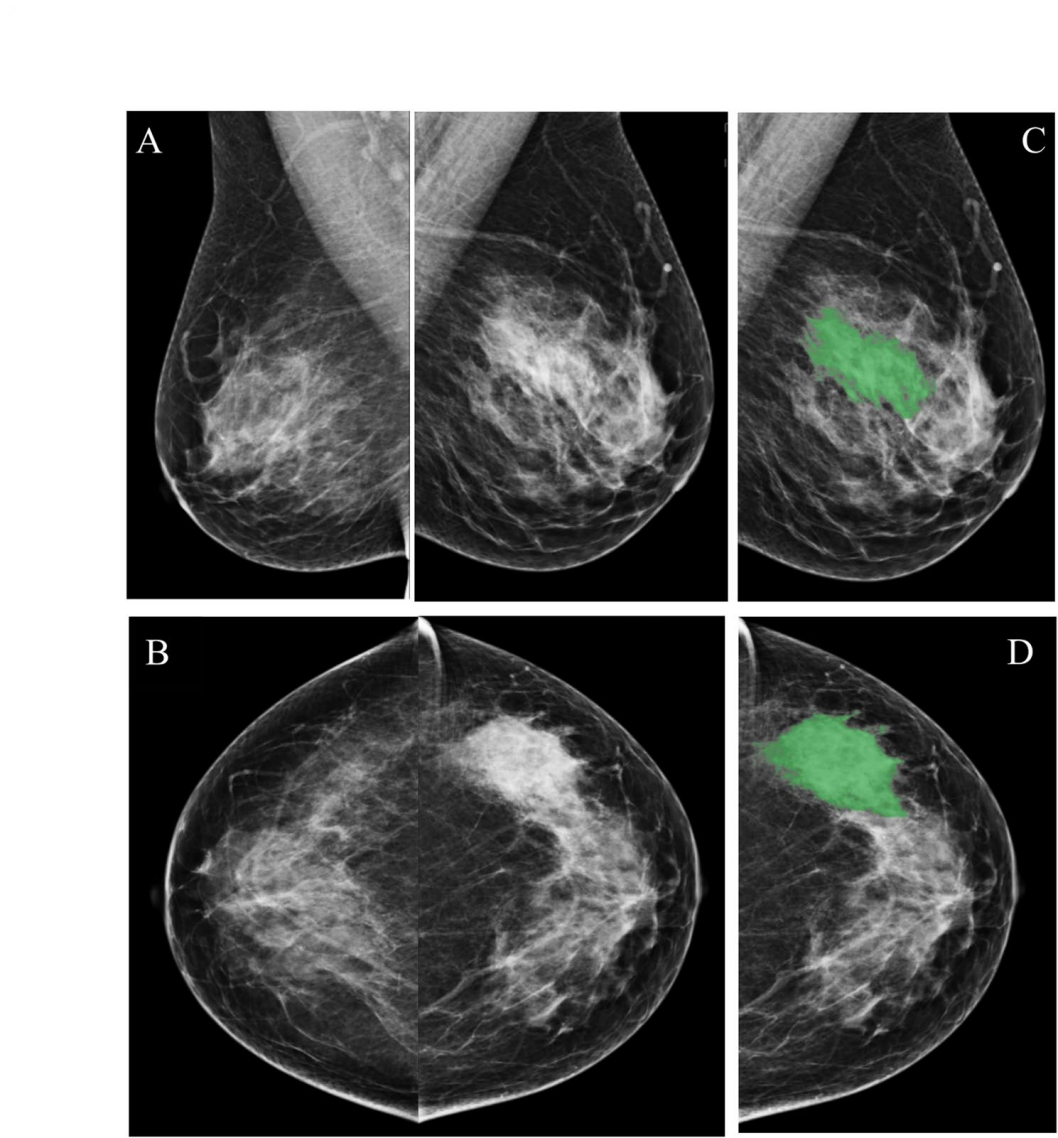
A 42-year-old woman presented with a localized area of dense tissue in the lateral region of the left breast and benign calcifications in the right breast. Mammographic images included the (**A**) MLO and (**B**) CC views, showing abnormalities in the left breast. Figures **C** and **D** show the ROI annotation of the lesion in this patient

**Fig. 3:**
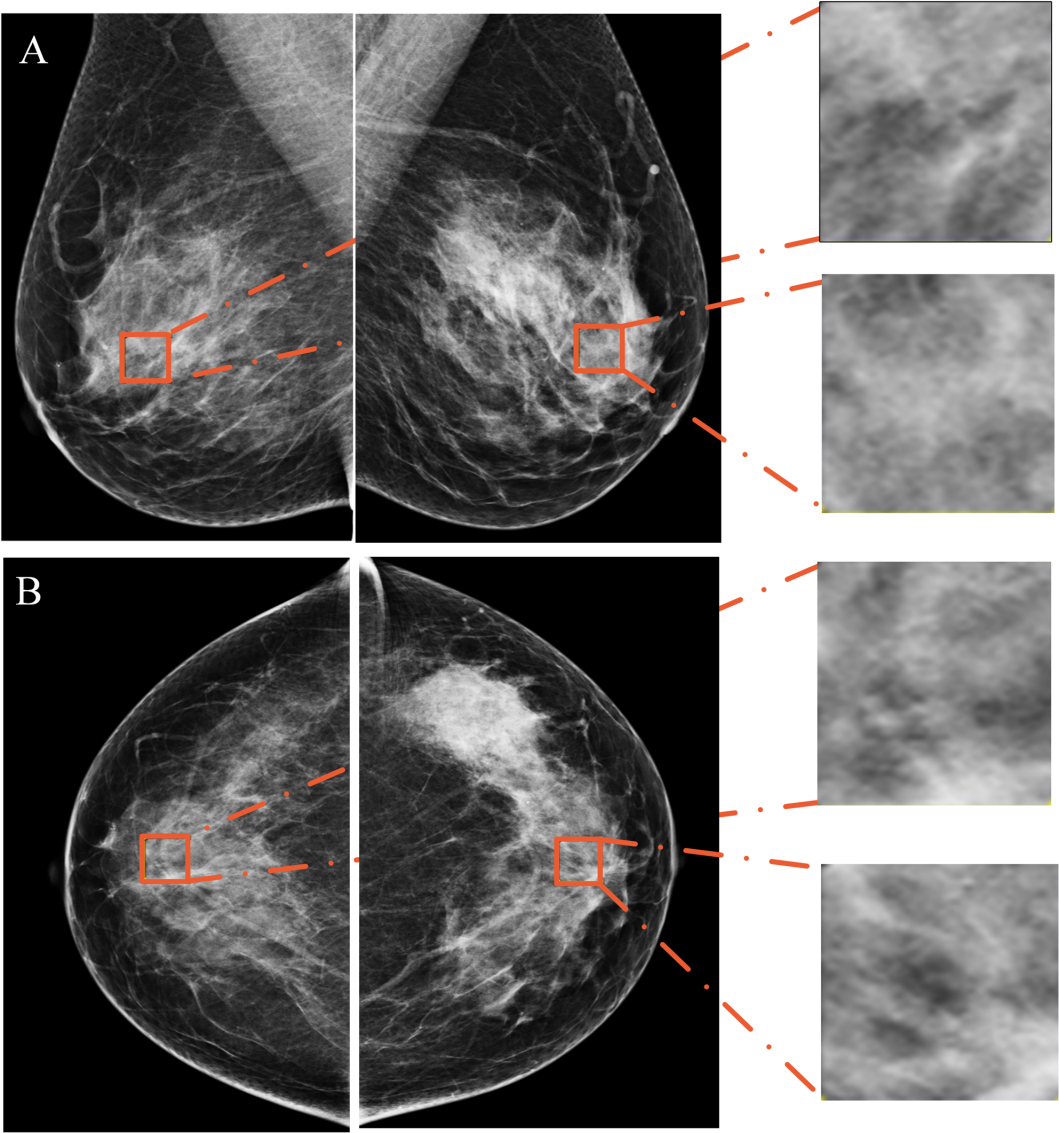
Figures** A** and **B** show the 1.5 cm × 1.5 cm square ROIs delineated in the retroareolar glandular region on both MLO and CC views, along with their corresponding magnified views

In the development cohort, all mammograms were acquired using a Selenia Dimensions™ digital mammography system (Hologic, USA) at Tianjin Medical University Cancer Institute & Hospital. In the external validation cohort, all mammograms were obtained using a Mammomat Inspiration system (Siemens Healthineers, Germany) at the First Affiliated Hospital of Bengbu Medical University. A semiautomatic segmentation module within the SIMPACS Research software was used to delineate tumors. The tumor regions of interest (ROIs) were delineated on both the mediolateral oblique (MLO) and craniocaudal (CC) views (Fig. 2). Mammography examinations were randomly assigned to two radiologists blinded to clinicopathologic data. Parenchymal analysis was performed bilaterally under the supervision of a breast radiologist. In both MLO and CC views, 1.5 cm×1.5 cm ROIs were manually placed by a junior radiologist in the central breast region immediately behind the nipple, targeting the densest parenchymal tissue while avoiding subcutaneous fat along the skin line (Fig. 3) [21]. All segmentation masks were confirmed by a senior radiologist (Yu Ji, with 10 years of experience in mammography).

### Feature selection and classification model Building

To analyze the radiomics dataset, a two-stage feature selection strategy was adopted to enhance interpretability and minimize overfitting. Initially, univariate statistical tests were performed: chi-square tests were used to identify significant categorical variables (*p* < 0.05), and Pearson correlation coefficients (PCC) were computed for continuous features. Highly correlated features (|PCC| > 0.8) were filtered to reduce multicollinearity. Subsequently, Least Absolute Shrinkage and Selection Operator (LASSO) regression, with 10-fold cross-validation was applied to select the most predictive features based on the 1-standard error criterion. The final subset of features obtained from LASSO was then used to construct several classification models. The final subset of features obtained from LASSO was then used to construct a multilayer perceptron (MLP) neural network, chosen as the primary classifier because of its ability to capture complex non-linear relationships in radiomic data. The MLP was trained exclusively in the development cohort, with its architecture and hyperparameters optimized by cross-validation. The final model was then retrained on the entire development cohort and subsequently evaluated in the external test cohort without further adjustment.

### Statistical analysis

Model performance was evaluated in both the development and external test cohorts using multiple metrics, including accuracy, area under the receiver operating characteristic curve (AUC), F1 score, precision, recall, and SHAP (SHapley Additive exPlanations) values. Accuracy and AUC quantified overall correctness and discriminative ability, while F1 score, precision, and recall were used to account for class imbalance. To assess model interpretability, we computed SHAP (SHapley Additive exPlanations) values for the ipsilateral radiomics–clinical MLP model and used SHAP summary plots to visualize the global contribution of each feature across all patients, with features ranked by their mean absolute SHAP values. Together, these evaluations provided a comprehensive assessment of predictive performance and generalizability across cohorts, as well as the interpretability of the model.

## Results

### Patient characteristics

A total of 121 patients were included in the study after applying the predefined inclusion and exclusion criteria at the two participating centers, with 81 patients in the development cohort (mean age, 53.5 ± 11.9 years) and 40 patients in the external test cohort (mean age, 50.4 ± 8.6 years). For both cohorts, PD-L1 expression was assessed using the 22C3 pharmDx assay, and the combined positive score (CPS) was calculated. With a CPS cut-off of 10, 26 of 81 patients (32.1%) in the development cohort and 24 of 40 patients (60.0%) in the external test cohort were classified as PD-L1 positive, whereas 55 (67.9%) and 16 (40.0%) cases, respectively, were classified as PD-L1 negative. The detailed clinicopathologic characteristics of the two cohorts are summarized in Table 1. Compared with the development cohort, the external test cohort showed a higher proportion of HER2 3 + tumors, ER-positive tumors, PR-positive tumors, and PD-L1–positive cases (all *p* < 0.01), whereas age and Ki-67 expression did not differ significantly between cohorts (Table 1). In the development cohort, there were no significant differences in age, HER2 status, Ki-67, ER, PR status, or histologic type between PD-L1–positive and PD-L1–negative tumors (all *p* > 0.05; Supplementary Table 1).

**Table 1:** Baseline clinicopathologic characteristics of the development and external test cohorts

Characteristics	Development cohort (*n* = 81)	External test cohort (*n* = 40)	*p*-value
**Age (years)**			
Mean ± SD	53.5 ± 11.9	50.4 ± 8.6	0.113
**HER2 status**,** n (%)**			**< 0.01**
0/1+	67 (82.7)	31 (77.5)	
2+	14 (17.3)	5 (12.5)	
3+	0 (0)	4 (10.0)	
**Ki-67**,** n (%)**			0.709
< 14%	6 (7.4)	6 (15.0)	
≥ 14%	75 (92.6)	34 (85.0)	
**ER**,** n (%)**			**< 0.01**
Negative	65 (80.2)	25 (62.5)	
Positive	16 (19.8)	15 (37.5)	
**PR**,** n (%)**			**< 0.01**
Negative	78 (96.3)	27 (67.5)	
Positive	3 (3.7)	13 (32.5)	
**Histologic type**,** n (%)**			–
NST	81 (100)	40 (100)	
Other	0 (0)	0 (0)	
**PD-L1 status**,** n (%)**			**< 0.01**
Positive	26 (32.1)	24 (60.0)	
Negative	55 (67.9)	16 (40.0)	

### Performance of the radiomics–clinical model for distinguishing PD-L1–positive versus –negative tumors

**Fig. 4:**
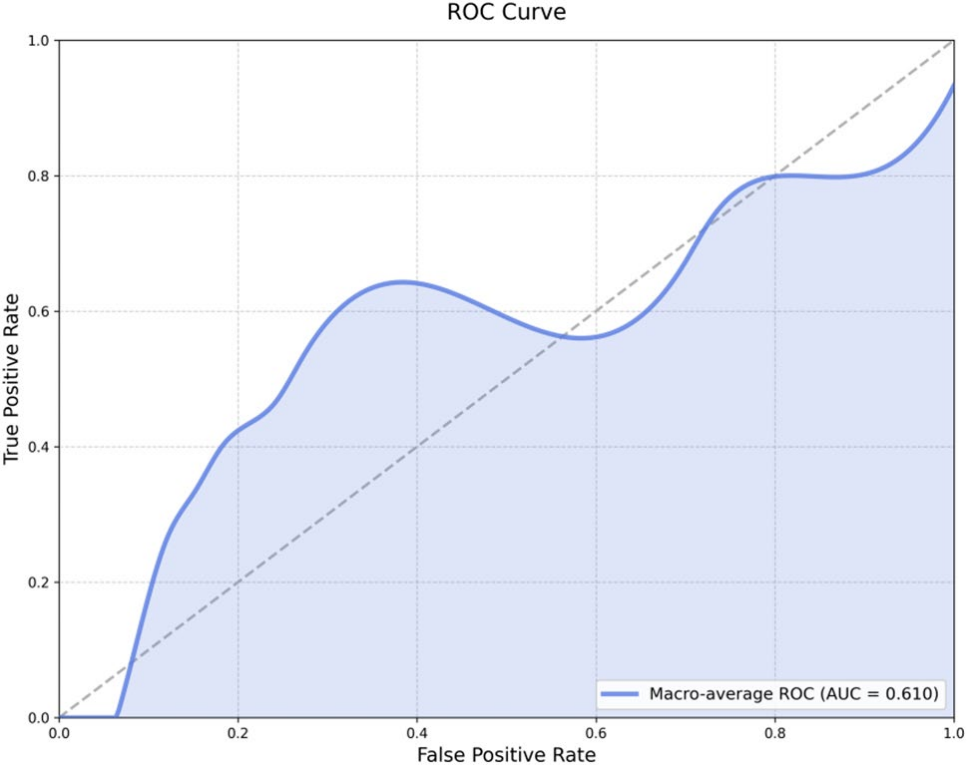
ROC curve of MLP model combining lesion and clinical features for PD-L1 prediction

The radiomic model incorporating clinicopathological information achieved an accuracy of 0.667 with an AUC of 0.610 (Fig. 4).

### Predictive performance of radiomics-based models with integrated mammographic background features

**Fig. 5:**
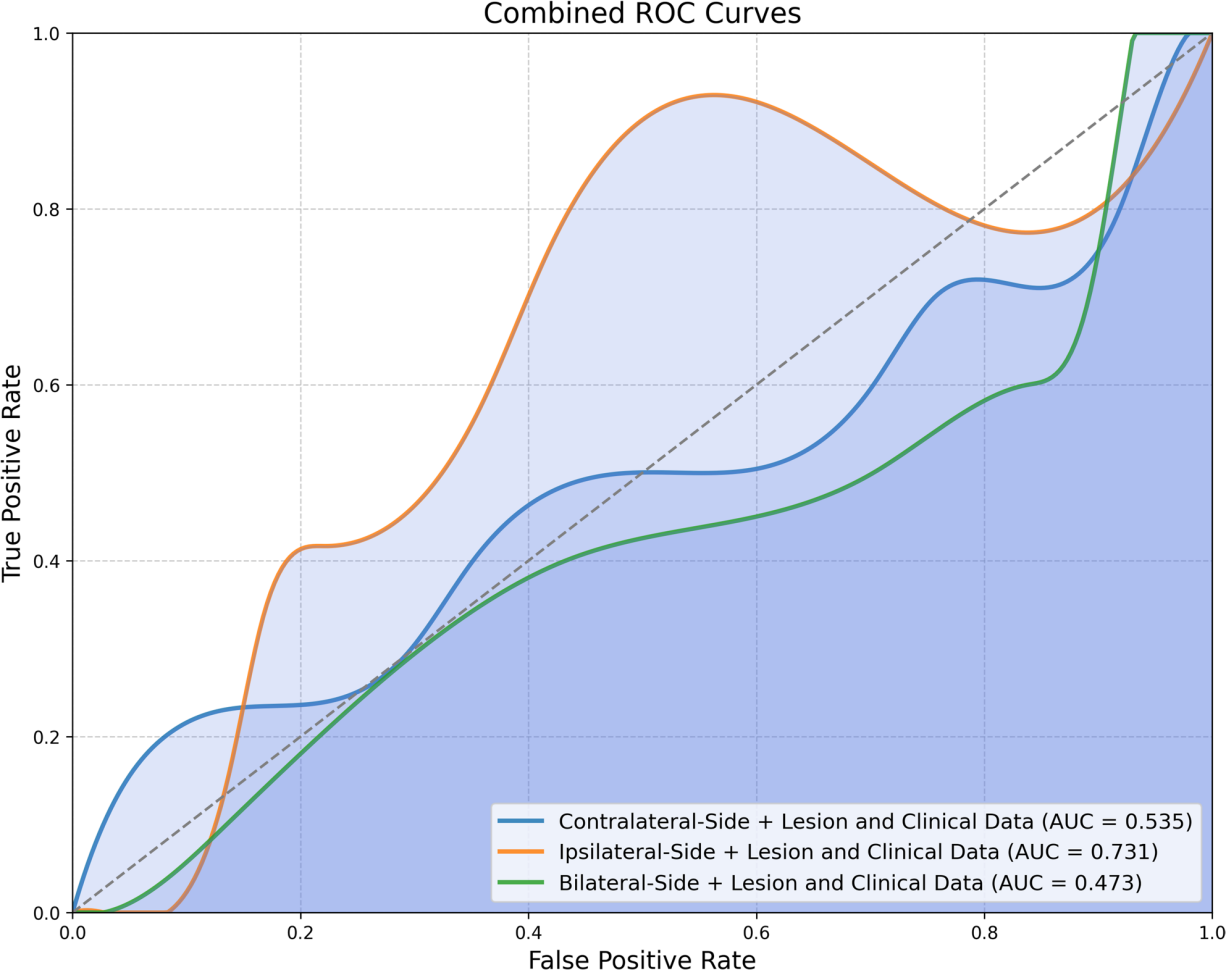
ROC curves of MLP models integrating lesion and clinical features with contralateral, ipsilateral, and bilateral breast density

When we evaluated the added value of incorporating mammographic breast density into PD-L1 prediction, the model combining lesion radiomic features, clinical data, and ipsilateral background density achieved significantly better performance than the model using lesion and clinical features alone (AUC = 0.731 vs. 0.610). This model achieved an accuracy of 0.708 and an F1 score of 0.587. The model integrating contralateral breast density with lesion radiomic features and clinical information achieved an AUC of 0.535, an accuracy of 0.681, and an F1 score of 0.540. The model incorporating bilateral breast density, along with lesion and clinical features, yielded an AUC of 0.537, an accuracy of 0.542, and an F1 score of 0.484 (Fig. 5). These findings suggest that ipsilateral parenchymal information may provide complementary diagnostic value, whereas contralateral features did not further enhance model performance in our dataset.

### External validation in the independent cohort

**Fig. 6:**
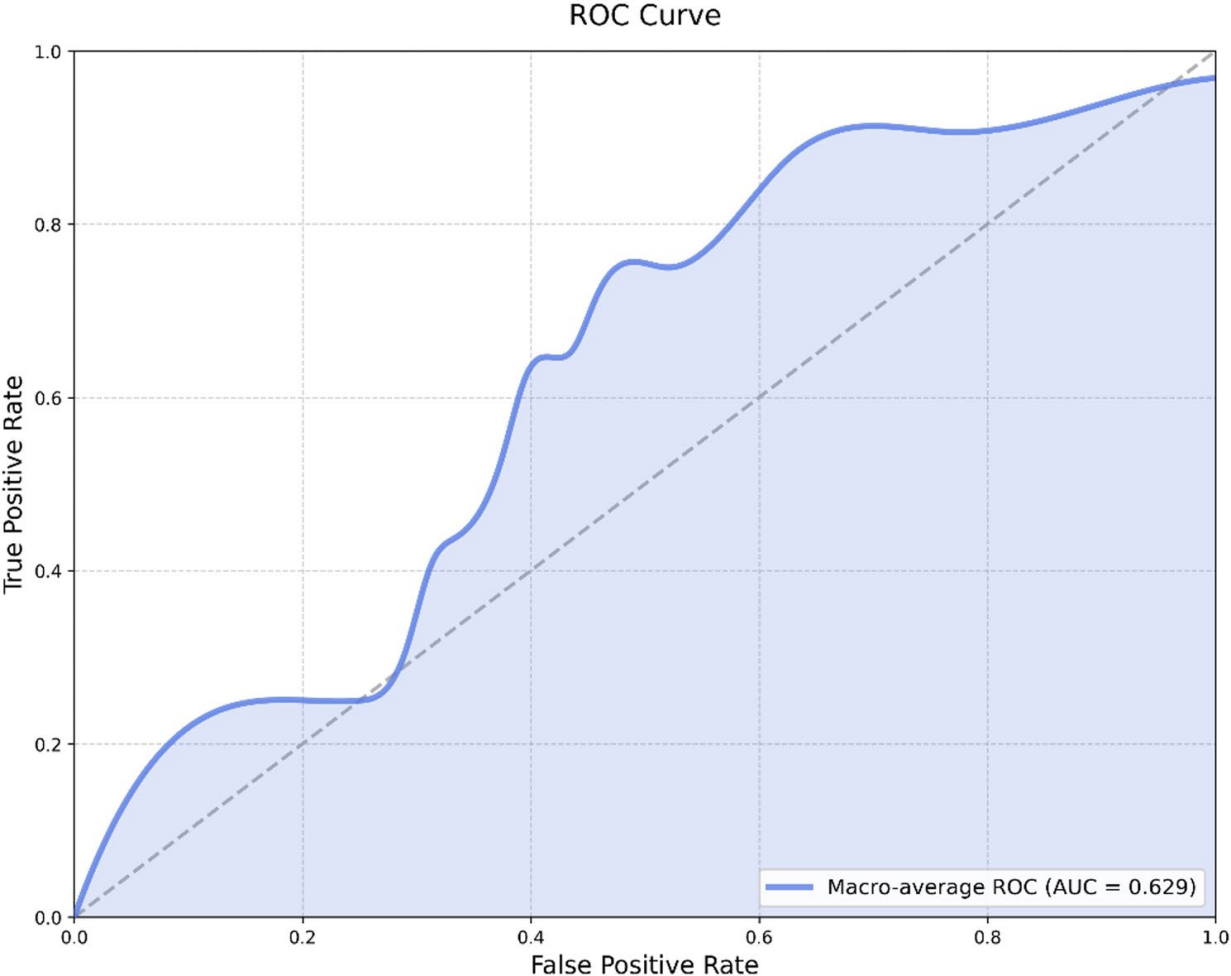
ROC curve of the ipsilateral radiomics–clinical MLP model in the external cohort (AUC = 0.629)

When the final ipsilateral radiomics–clinical MLP model was applied to the independent external cohort without recalibration, it achieved an AUC of 0.629 (Fig. 6), indicating modest discrimination for PD-L1 prediction.

### Model interpretability based on SHAP analysis

**Fig. 7:**
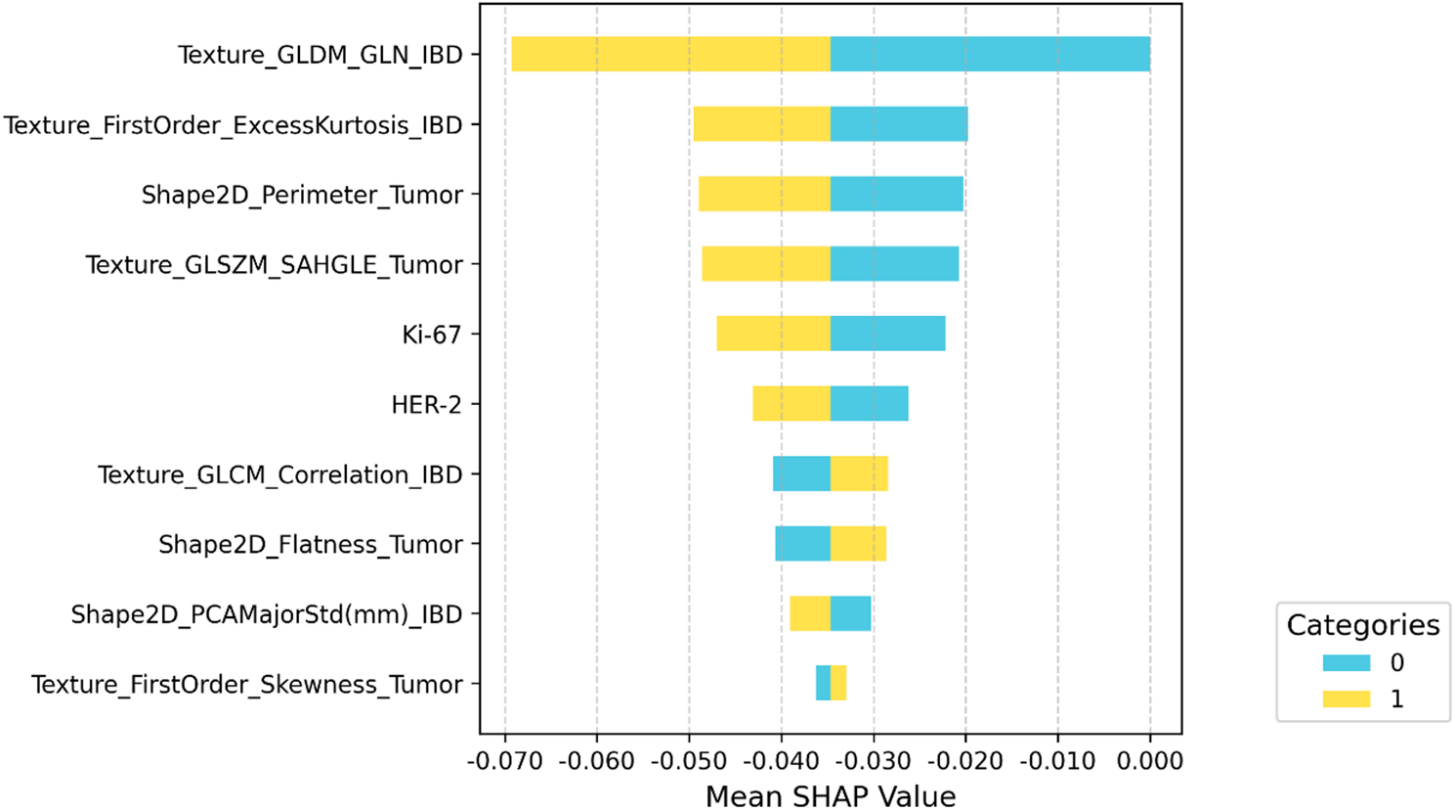
SHAP summary plot for the ipsilateral radiomics–clinical MLP model. Bars show mean absolute SHAP values for the top 10 features; suffixes indicate feature origin (“_Tumor” for tumor radiomics and “_IBD” for ipsilateral breast density)

To assess model interpretability, we examined SHAP values of the ipsilateral radiomics–clinical MLP model in the development cohort (Fig. 7). The SHAP summary plot showed that texture features from the ipsilateral breast density and tumor shape/texture descriptors were the dominant contributors to PD-L1 prediction. Ipsilateral background features (e.g., Texture_GLDM_GLN_IBD and Texture_FirstOrder_ExcessKurtosis_IBD) and tumor-based features (e.g., Shape2D_Perimeter_Tumor and Texture_GLSZM_SAHGLE_Tumor) had the highest mean absolute SHAP values, while Ki-67 and HER-2 also exhibited notable contributions. Overall, the SHAP analysis indicates that ipsilateral background and tumor radiomics features, together with key clinicopathologic variables, jointly drive the model’s prediction of PD-L1 expression.

## Discussion

This study demonstrated that mammography-based radiomics models can effectively predict PD-L1 expression in breast cancer, with the AUC improving from 0.626 to 0.731 after incorporating ipsilateral breast density. These findings suggest the added value of ipsilateral parenchymal background information in PD-L1 assessment. In an independent external cohort, the ipsilateral radiomics-clinical MLP model yielded an AUC of 0.629, providing preliminary evidence of external validity while indicating that further refinement and validation are required.

The improved performance may stem from radiomics’ ability to capture tumor heterogeneity and subtle image changes undetectable by clinical assessment. Similar findings have been reported in MRI-based radiomics studies for biomarker prediction. Our results align with those of Hu et al. (2024), who showed that MRI-derived radiomics features could predict PD-L1 expression with high accuracy [22]. Unlike their study, we utilized mammography, which is more widely available and cost-effective, making it more suitable for routine clinical use.

Breast tumors exhibit significant heterogeneity, with spatial variations in PD-L1 expression across different tumor regions. Conventional biopsy-based assessments often fail to capture this variability. Small tissue samples may not accurately represent the overall tumor microenvironment. Discrepancies between biopsy and surgical specimens highlight the limitations of this approach, with biopsies potentially underestimating or misrepresenting PD-L1 expression [23, 24]. Variability in PD-L1 detection assays, such as SP142 and 22C3, further complicates assessment. Each platform uses distinct criteria, targeting different cell types (tumor vs. immune cells) and scoring systems (e.g., IC scores vs. CPS), leading to inconsistencies in patient classification and treatment decisions [19]. Radiomics provides a non-invasive, standardized method to assess PD-L1 expression by analyzing entire tumor volumes. This may help overcome biopsy limitations and platform variability to enhance patient selection for immune therapies.

From a clinical perspective, our mammography-based radiomics model should currently be regarded as an exploratory tool that may assist, rather than replace, tissue-based PD-L1 immunohistochemistry, which remains the reference standard. In patients who already undergo mammography, an imaging-derived estimate of PD-L1 positivity could help identify individuals who are more likely to benefit from confirmatory PD-L1 testing and immunotherapy, and may be particularly helpful when biopsy is technically challenging or yields limited tissue. Thus, this approach may help to optimize and complement invasive PD-L1 assessment, although its actual impact on treatment decision-making needs to be confirmed in future prospective studies.

We observed that incorporating ipsilateral breast density improved model performance, while including contralateral density reduced it. Prior studies have established that higher mammographic density is an independent risk factor for breast cancer [25–27], largely due to increased fibroglandular tissue, epithelial proliferation, and stromal remodeling [28]. Notably, density is not purely systemic but can vary locally. Ipsilateral parenchyma may exhibit subtle tumor-induced changes, such as fibrosis, inflammation, and altered architecture.These changes may elevate local density and create distinct textural patterns detectable by radiomics. This biologically plausible link may explain why incorporating ipsilateral density improved PD-L1 prediction, reflecting its association with the tumor immune microenvironment. In contrast, contralateral density likely reflects systemic background rather than local tumor biology, which may add non-specific noise and reduce model performance. This is consistent with the understanding that mammary parenchyma and tumor microenvironment play a key role in cancer progression and immune regulation [29].

In line with this concept, our SHAP-based analysis showed that texture descriptors of the ipsilateral breast density (Texture_GLDM_GLN_IBD and Texture_FirstOrder_ExcessKurtosis_IBD) ranked among the top features, while tumor-derived shape and texture metrics such as Shape2D_Perimeter_Tumor and Texture_GLSZM_SAHGLE_Tumor also contributed substantially, indicating that tumor-side parenchymal patterns, boundary complexity and small high-gray-level foci encode PD-L1-related heterogeneity; similar shape descriptors and GLSZM-based texture features have been associated with immune cell infiltration, tumor microenvironment phenotypes, or response to PD-1/PD-L1 blockade in previous radiomics studies. Although Ki-67 and HER-2 were not significantly associated with PD-L1 status in our univariate analysis, prior work has linked proliferation markers and HER2-positive breast cancers with PD-L1 expression and more aggressive clinicopathologic features [30, 31], and in our model both variables still showed non-negligible SHAP contributions, suggesting that proliferation and molecular subtype may provide complementary information when integrated with tumor and ipsilateral background radiomics.

This study has several limitations. First, the overall sample size, particularly the number of PD-L1–positive cases, was modest. Second, external validation showed only moderate discrimination (AUC = 0.629), which may be related to differences in case mix, imaging protocols, and clinicopathologic profiles (including HER-2 status, hormone receptor expression, and PD-L1 positivity) between centers, thereby limiting model transferability. Third, the model was built solely on mammographic data; integration of additional imaging modalities may further improve performance. Future prospective studies with larger, more diverse multicenter cohorts are needed to refine the model and rigorously evaluate its clinical utility.

## Conclusion

Mammography-based radiomics combined with clinicopathological data may enable effective prediction of PD-L1 expression in breast cancer. Including ipsilateral breast density improved model performance and underscored the contribution of tumor-side parenchymal information to PD-L1–related imaging phenotypes. The ipsilateral radiomics–clinical MLP model was further evaluated in an independent external cohort, providing preliminary evidence of external validity. These findings suggest that such models may support immunotherapy decision-making, although further refinement and validation in larger multicenter populations are required before clinical implementation.

## Supplementary Information

Below is the link to the electronic supplementary material.

Supplementary Material 1

## Data Availability

The datasets generated and/or analyzed during the current study (including mammography DICOM images, ROI/segmentation masks, extracted radiomic features, and clinicopathologic variables) contain identifiable patient information and are therefore not publicly available due to institutional and ethical restrictions. De-identified data may be made available from the corresponding author upon reasonable request and with approval from the ethics committees of Tianjin Medical University Cancer Institute & Hospital and The First Affiliated Hospital of Bengbu Medical University, and subject to a data use agreement.

## Abbreviations

PD: L1 Programmed death-ligand 1
CPS: Combined Positive Score
MLP: Multilayer perceptron
MLO: Mediolateral oblique
CC: Craniocaudal
LASSO: Least absolute shrinkage and selection operator
AUC-ROC: Area under the receiver operating characteristic curve
ER: Estrogen Receptor
PR: Progesterone Receptor
HER2: Human Epidermal Growth Factor Receptor 2
Ki-67: Ki-67 Proliferation Index
