# Supplementary material for: Mammographic radiomics and breast density for predicting PD-L1 expression in breast cancer
Source: Cancer Imaging. 2026 Feb 7;26:38. doi: 10.1186/s40644-026-01001-3 (PMC12977622; doi:10.1186/s40644-026-01001-3)
Supplement: Supplementary file 1 — Supplementary Material 1 [file 40644_2026_1001_MOESM1_ESM.docx]

Supplementary Table 1. Clinicopathological characteristics of the PD-L1-positive set and negative set breast cancer tumors included in this study

|  | PD-L1-positive  （n=26） | PD-L1- negative  （n=55） | *p*-value |
| --- | --- | --- | --- |
| Age (years)  Mean | 50.1 ± 13.6 | 52.3 ± 11.2 | 0.636 |
| HER2  0/1+  2+  3+ | 21 (80.8%)  5(19.2%)  0 (0%) | 46 (83.6%)  9 (16.4%)  0 (0%) | 0.670 |
| Ki-67  < 14%  ≥ 14% | 23 (88.5%)  3 (11.5%) | 52 (94.5%)  3 (5.5%) | 0.449 |
| ER  Negative  Positive | 19 (73.1%)  7(26.9%) | 46 (83.7%)  9(16.3%) | 0.094 |
| PR  Negative  Positive | 25 (96.2%)  1 (3.8%) | 53 (96.4%)  2 (3.6%) | 0.835 |
| Histology type  NST  Other | 26  0 | 55  0 | - |

HER2, human epidermal growth factor receptor 2; ER, estrogen receptor; PR, progesterone receptor; NST, invasive carcinoma of no special type.
